# Polyglutamine-expanded ataxin3 alter specific gene expressions through changing DNA methylation status in SCA3/MJD

**DOI:** 10.18632/aging.202331

**Published:** 2020-12-19

**Authors:** Dongxue Ding, Chunrong Wang, Zhao Chen, Kun Xia, Beisha Tang, Rong Qiu, Hong Jiang

**Affiliations:** 1 Department of Neurology, Xiangya Hospital, Central South University, Changsha, Hunan, P.R. China; 2 State Key Laboratory of Medical Genetics, Central South University, Changsha, Hunan, P.R. China; 3 Key Laboratory of Hunan Province in Neurodegenerative Disorders, Central South University, Changsha, Hunan, P.R. China; 4 National Clinical Research Center for Geriatric Diseases, Changsha, Hunan, P. R. China; 5 School of Information Science and Engineering, Central South University, Changsha, China

**Keywords:** SCA3/MJD, DNA methylation, RRBS, gene expression, dynamic methylation

## Abstract

DNA methylation has recently been linked to transcriptional dysregulation and neuronal dysfunction in polyglutamine (polyQ) disease. This study aims to determine whether (CAG)_n_ expansion in *ATXN3* perturbs DNA methylation status and affects gene expression. We analyzed DNA methylation throughout the genome using reduced representation bisulfite sequencing (RRBS) and confirmed the results using MethylTarget sequencing. Dynamic changes in DNA methylation, transcriptional and translational levels of specific genes were detected using BSP, qRT-PCR and western blot. In total, 135 differentially methylated regions (DMRs) were identified between SCA3/MJD and WT mouse cerebellum. KEGG analysis revealed differentially methylated genes involved in amino acid metabolism, Hedgehog signaling pathway, thyroid cancer, tumorigenesis and other pathways. We focused on DMRs that were directly associated with gene expression. On this basis, we further assessed 7 genes, including 13 DMRs, for DNA methylation validation and gene expression. We found that the methylation status of the DMRs of *En1* and *Nkx2-1* was negatively associated with their transcriptional and translational levels and that alteration of the DNA methylation status of DMRs and the corresponding transcription occurred before dyskinesia in SCA3/MJD mice. These results revealed novel DNA methylation-regulated genes, *En1* and *Nkx2-1*, which may be useful for understanding the pathogenesis of SCA3/MJD.

## INTRODUCTION

Polyglutamine (polyQ) expansion diseases are a group of hereditary neurodegenerative disorders, including spinobulbar muscular atrophy (SBMA), Huntington's disease (HD), dentatorubral-pallidoluysian atrophy (DRPLA) and six forms of spinocerebellar ataxia (SCA1, 2, 3, 6, 7 and 17) [1, 2]. These disorders share common pathogenesis in that all are caused by an unstable CAG expansion mutation in a causative gene that leads to an expanded polyQ tract in the corresponding protein [1–4]. Accumulating data suggest that the expanded polyQ tract triggers a pathogenic cascade, leading to selective neuronal cell dysfunction and death [1–4]. However, the precise pathogenic mechanism of neurodegeneration triggered by polyQ proteins remains largely unknown.

As a representative polyQ disease, SCA3/MJD is the most prevalent ataxia transmitted via dominant inheritance worldwide [5]. It is caused by an abnormal CAG expansion in exon 10 of *ATXN3*, leading to an expanded polyQ tract (55–87 glutamines in contrast to the normal 10-51) near the C-terminus of the mutant ataxin-3 protein [6, 7]. This expanded protein could result in selective neuronal loss, predominantly in the brainstem, cerebellum (spinocerebellar pathways and dentate nucleus), striatum, thalamus, substantia nigra and pontine nuclei. Because of this, SCA3/MJD is characterized by progressive ataxia, oculomotor abnormalities, dystonia and peripheral neuropathy [3, 4]. Furthermore, the severity of symptoms and the age at onset (AAO) of SCA3/MJD are positively and negatively related to the CAG expansion length in *ATXN3*, respectively [8, 9]. Although SCA3/MJD is monogenic, as it arises from a mutation in *ATXN3*, this disease is highly complex, and its pathogenic mechanism has not yet been fully elucidated.

Pathologically, transcriptional dysregulation caused by polyQ expansion is thought to be a major contributor to selective neuronal death in SCA3/MJD [10]. Intranuclear accumulation of mutant ataxin3 and transcription factor recruitment indicate that transcriptional dysfunction may play an important role in the pathogenesis of SCA3/MJD [11]. Chou’s team detected transcriptional changes in cultured cerebellar and substantia nigra neurons [12] and in transgenic SCA3/MJD mice [13]. In addition, they found that expanded ataxin3 could activate the mitochondrial apoptotic pathway and induce neuronal death by regulating gene expression [12]. In recent years, alterations in gene expression levels have also been detected in peripheral blood samples from SCA3/MJD patients [14]. However, the underlying molecular mechanism by which expanded ataxin3 affects gene expression remains unclear. DNA methylation, the most extensively studied epigenetic phenomenon, can alter the chromosomal stability and gene expression of an organism and is one of the major DNA modifications essential for the regulation of gene transcription and genomic function [15]. It can affect gene expression at the transcriptional and posttranscriptional levels by either interfering with the binding of transcription factors to promoters or altering mRNA processing [16]. In recent years, changes in DNA methylation have been detected in several neurodegenerative diseases, such as Parkinson’s disease [17], Alzheimer's disease [18], SCA2 [19], and SCA7 [20]. Our previous study suggested that DNA methylation in the promoter of *ATXN3* might influence the AAO and progression of SCA3/MJD patients [21]. Additionally, we demonstrated that polymorphisms in DNA methylation-related genes are associated with CAG distribution and AAO in SCA3/MJD patients [22]. However, little information is available on whether and how DNA methylation contributes to transcriptional dysfunction in SCA3/MJD.

In this research, we hypothesized that aberrant DNA methylation in specific genes is an underlying cause of transcriptional dysfunction in SCA3/MJD neurons. We performed reduced representation bisulfite sequencing (RRBS), a cost-efficient method of genome-wide DNA methylation analysis, to test the DNA methylation levels in the cerebellum of the SCA3/MJD mouse model. Transcriptional changes were also measured by qRT-PCR to study the relationship between DNA methylation and gene expression in SCA3/MJD. The data showed that expanded ataxin3 could perturb the status of DNA methylation and then alter the transcription of En1 and Nkx2-1 in the SCA3/MJD mouse model. Changes in DNA methylation and gene expression occurred before the appearance of ataxia symptoms in SCA3/MJD mice. Together, these results indicate that abnormal (CAG)n expansion in *ATXN3* could influence the DNA methylation status and transcript levels of specific genes. Therefore, the data suggest that the novel DNA methylation-regulated genes *En1* and *Nkx2-1* may be involved in the pathogenesis of SCA3/MJD.

## RESULTS

### Symptoms in mice

**Figure 1:**
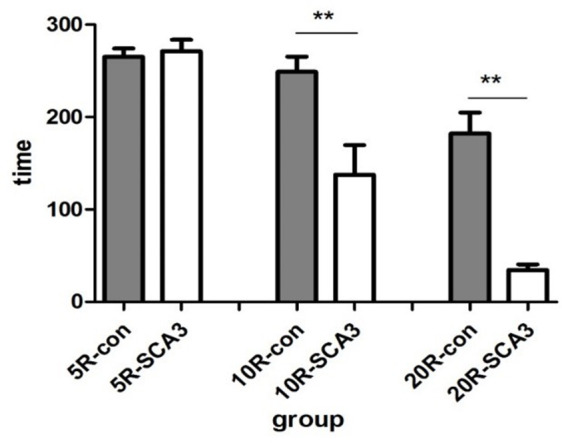
**SCA3/MJD mice fail in the accelerated rotarod test.** All animals managed to stay on a rod rotating at constant speed (5rpm) for about 5 min. When the rotation was accelerated to 10 rpm and 20 rpm, latency to fall from the test apparatus was significantly reduced in SCA3/MJD mice (*p*<0.05). And the extent of reduction is depended on the rotating speed. When the speed reached 20 rpm, the latency to fall in SCA3/MJD mice were about 1min.

In this study, approximately 50% of the mice in the second generation expressed disease-causing human ataxin-3 with an expanded polyglutamine tract, similar to the report from the Jackson laboratory [23]. The number of CAG repeats in the expanded *ATXN3* was 83 or 84 in the second-generation SCA3/MJD mice. To observe their symptoms, the rotation test was conducted. The ataxin3-83/84Q mice exhibited impaired motor coordination and a significantly shorter latency to fall than the WT mice at the same age of 32 weeks (Figure 1). In addition, the ataxin3-83/84Q mice displayed an ataxic, wide-based and irregular gait compared to that of WT mice at 32 weeks (data not shown). Overall, these results indicated that ataxic symptoms similar to those of SCA3 clearly appeared in ataxin3-83/84Q mice at the age of 32 weeks.

### DNA methylation status in 32-week-old mice

**Figure 2:**
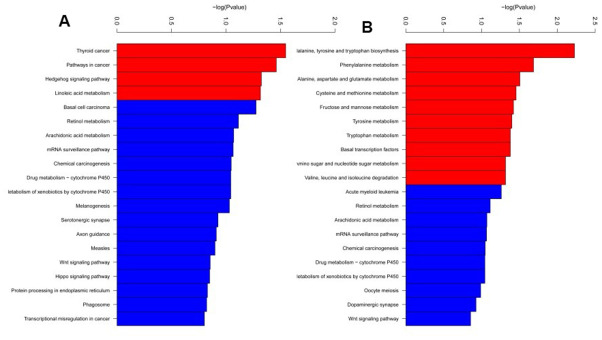
**KEGG pathway enrichment analysis of differentially methylated regions.** Totally 14 pathways were enriched. (**A**) KEGG pathway enriched from the hypermethylated regions in SCA3/MJD mice compared to controls. (**B**) KEGG pathways enriched from the hypomethylated regions in SCA3/MJD mice compared to controls. The major pathways were involved in the pathway for tumorigenesis and amino acid biosynthesis.

RRBS was carried out using 3 SCA3/MJD and 3 WT mice to identify differentially methylated CpG sites and regions. The average global CpG methylation levels of RRBS samples were 55.86 ±1.1% and 58.07±1.8% in WT and SCA3/MJD group, respectively. No significant difference on global DNA methylation was detected (*p*>0.05). After quality control and analysis of the data, a total of 135 DMRs were identified between the two groups. As shown in Supplementary Tables 6 and 7, 62 DMRs were hypomethylated and 73 were hypermethylated in SCA3/MJD mice compared to WT mice. Supplementary Tables 8 and 9 showed the DNA methylation levels of CpG sites of DMRs in WT and SCA3/MJD groups. GO and KEGG enrichment analyses were applied to evaluate the differentially methylated genes. In the GO functional analysis, the hypomethylated DMRs in SCA3/MJD mice were enriched in 400 major functional groups. Among these functional groups, 356 were categorized as biological process, only 1 as cellular component, and 40 as molecular function. Among the hypermethylated DMRs in SCA3/MJD mice, 348 major functional groups were enriched. Likewise, 254 groups were assigned to biological process, 23 to cellular component, and 71 to molecular function. As shown in Figure 2, according to the KEGG enrichment analysis, the DMRs were involved in 14 pathways. More specifically, the major DMRs were involved in the pathway tumorigenesis and amino acid biosynthesis.

### Validation of methylation results and detection of transcription of the identified markers

From all the genes with identified DMRs, 7 genes containing 13 DMRs were reported associated with the function of neurons: *En1*, *Fbxo41*, *Nkx2-1*, *Syngr1*, *Rara*, *Otx1*, and *Frzb*. To determine whether these candidate genes were differentially expressed in WT and SCA3/MJD mouse cerebellum, we analyzed their mRNA expression with qRT-PCR. And their DNA methylation levels were confirmed using MethylTarget sequencing. The primers used for MethylTarget verification and the qRT-PCR are shown in Supplementary Tables 3, 4.

**Figure 3:**
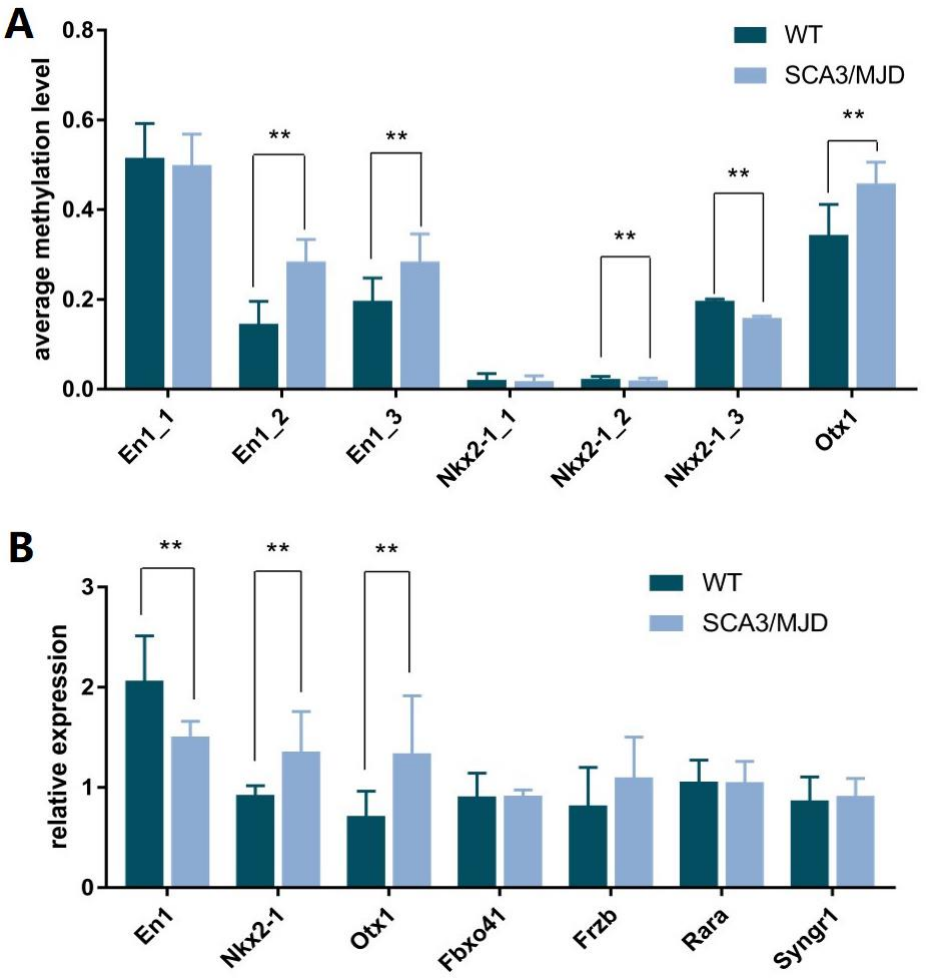
**DNA methylation status of sequence in *En1*, *Nkx2-1*, and *Otx1* and the corresponding transcriptional levels of these genes.** (**A**) Compared to control group, the average methylation levels of sequence En1_2, En1_3, and Otx1 were higher in SCA3/MJD mice (*p*<0.05). While the average methylation levels of sequences Nkx2-1_2 and Nkx2-1_3 were lower in SCA3/MJD mice (*p*<0.05). (**B**) the transcriptional levels of *En1*, *Nkx2-1* and *Otx1* were significantly different between SCA3/MJD and control groups (*p*<0.05). No significantly differences were detected in the other genes.

**Figure 4:**
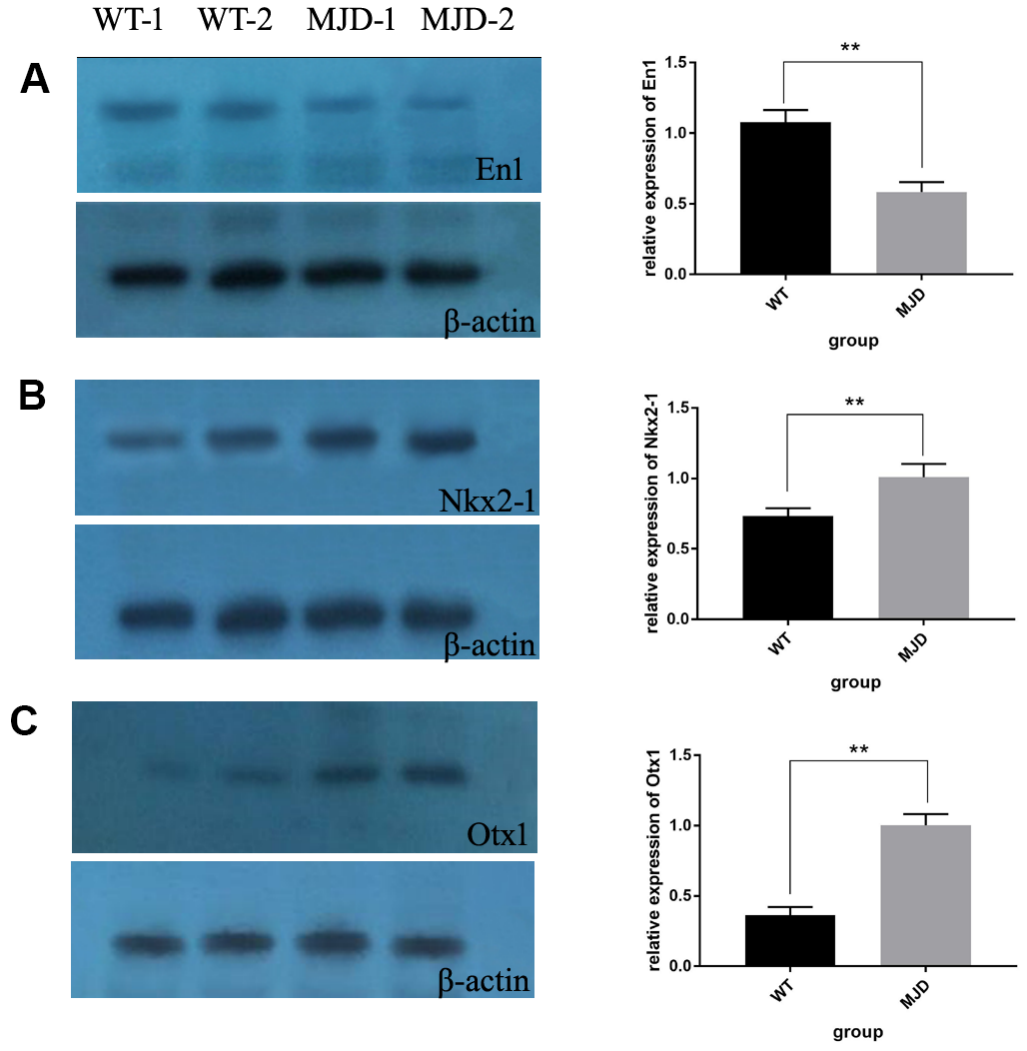
**Protein levels of En1, Nkx2-1, and Otx1 in 32-week old mice cerebellum.** (**A**) Western blot analysis of En1 in both control and SCA3/MJD groups. Compared to control group, the protein level of En1 was significantly downregulated in SCA3/MJD group (*p*<0.05). (**B**) Western blot analysis of Nkx2-1 in both control and SCA3/MJD groups. Compared to control group, the protein level of Nkx2-1 was significantly upregulated in SCA3/MJD group. (**C**) Western blot analysis of Otx1 in both control and SCA3/MJD groups. Compared to control group, the protein level of Otx1 was significantly upregulated in SCA3/MJD group.

In total, 12 cerebellum samples from 6 SCA3/MJD and 6 WT mice of 32-week old were used for the validation experiments. As shown in Table 1 and Figure 3A, compared to the WT group, the En1 (En1_2 and En1_3) and Otx1 (Otx1) sequences were significantly hypermethylated in the SCA3/MJD group. By contrast, significant hypomethylation was detected for Nkx2-1 (Nkx2-1_2 and Nkx2-1_3) in the SCA3/MJD group. As shown in Table 2 and Figure 3B, significant differences in mRNA levels were detected in *En1*, *Nkx2-1* and *Otx1*, which exhibited distinct DNA methylation patterns in the two groups. Compared to those in the WT group, the mRNA levels of *Nkx2-1* and *Otx1* were upregulated in SCA3/MJD group, while *En1* was downregulated. Therefore, we chose *En1*, *Otx1* and *Nkx2-1* for the next analysis. To further consolidate the qRT-PCR results, we compared the translational level of *En1*, *Otx1* and *Nkx2-1* between SCA3/MJD and WT mice by western blot. As shown in Figure 4, consistent with the results of mRNA levels, the protein level of *En1* was downregulated while the protein levels of *Otx1* and *Nkx2-1* were upregulated in SCA3/MJD mice (*p*<0.05).

**Table 1:** Rate of DNA methylation in selected sequence of SCA3/MJD and WT mice.

**Gene**	**Sequence**	**Control**	**SCA3/MJD**	***p* value**
*En1*	En1_1	0.516 ± 0.076	0.500±0.069	0.651
	En1_2	0.146 ± 0.050	0.285±0.049	0.045*
	En1_3	0.197 ± 0.051	0.285±0.061	0.007*
*Fbxo41*	Fbxo41	0.205±0.067	0.196±0.066	0.617
*Frzb*	Frzb_1	0.018±0.014	0.017 ±0.012	0.715
	Frzb_2	0.177±0.015	0.151±0.011	0.550
*Nkx2-1*	Nkx2-1_1	0.021±0.014	0.018± 0.012	0.552
	Nkx2-1_2	0.023 ±0.006	0.020±0.005	0.049*
	Nkx2-1_3	0.197±0.004	0.159±0.004	0.013*
*Rara*	Rara_1	0.433±0.140	0.434±0.159	0.998
	Rara_2	0.414±0.115	0.447±0.129	0.754
*Syngr1*	Syngr1_1	0.387±0.223	0.383±0.226	0.961
*Otx1*	Otx1	0.344±0.068	0.459±0.047	0.007*

*: *p*<0.05.

**Table 2:** The relative expression of selected genes in SCA3/MJD and WT mice.

**Gene**	**Control (n=6)**	**SCA3/MJD (n=6)**	***p* value**
*Otx1*	0.717 ± 0.247	1.340 ± 0.576	0.035*
*En1*	2.067 ± 0.446	1.509 ± 0.151	0.018*
*Nkx2-1*	0.926 ± 0.092	1.358 ± 0.401	0.028*
*Fbxo41*	0.911 ± 0.233	0.919 ± 0.083	0.057
*Frzb*	0.820 ± 0.381	1.101 ± 0.402	0.657
*Rara*	1.058 ± 0.215	1.053 ± 0.209	0.897
*Syngr1*	0.871 ± 0.234	0.915 ± 0.177	0.735

*: *p*<0.05.

### Dynamic changes in DNA methylation status and transcript level

**Figure 5:**
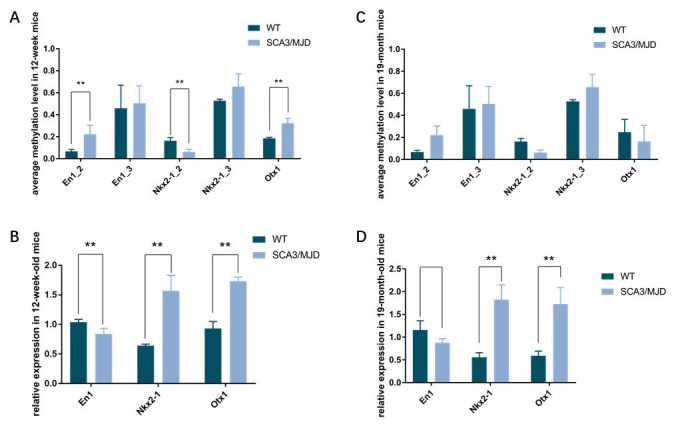
**Dynamic DNA methylation and gene expression change of *En1*, *Nkx2-1*, and *Otx1.*** (**A**, **B**) the average DNA methylation levels and transcriptional levels in 12-week-old mice. DNA methylation levels of sequence En1_2, Nkx2-1_2 and Otx1were significantly different between the two groups (*p*<0.05). In 12-week-old mice, expression of *En1*, *Nkx2-1* and *Otx1* were significantly different in the two groups (*p*<0.05). (**C**, **D**) the average DNA methylation levels and transcriptional levels in 19-month-old mice. No significant difference was observed for DNA methylation levels in sequences in *En1*, *Nkx2-1* and *Otx1*. Expression of *Nkx2-1* and *Otx1* were still significantly different in the two groups (*p*<0.05).

**Figure 6:**
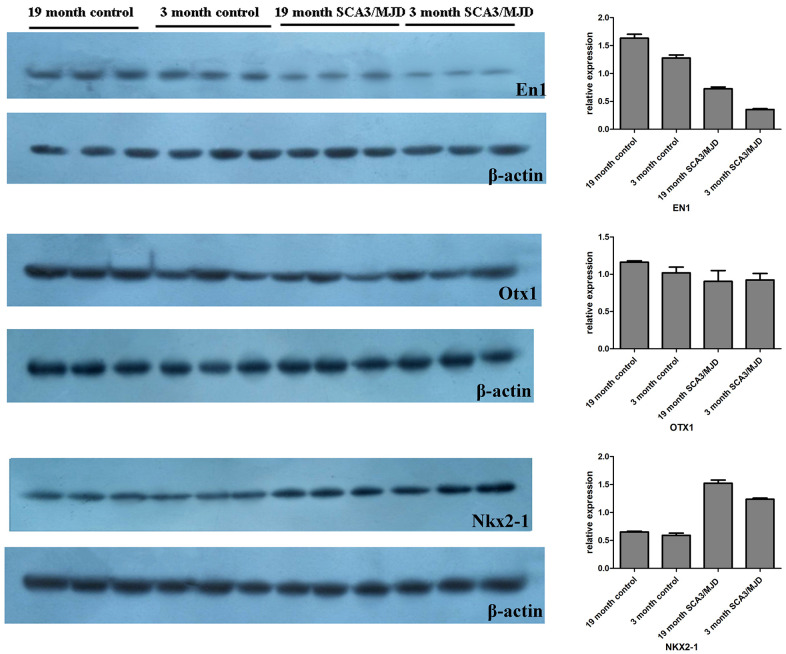
**Dynamic changes of *En1*, *Nkx2-1*, and *Otx1* protein levels in 12-week and 19-month old mice cerebellum.** Compared to the WT group, the protein level of En1 showed a significant downregulation in SCA3/MJD group at both 12 weeks and 19 months (*p*<0.05). And at 19 months of age, the protein level of En1 was higher than that at 12 weeks in both WT and SCA3/MJD mice cerebellum (*p*<0.05). For Nkx2-1, the protein levels were significantly upregulated in the SCA3/MJD group than in WT group (*p*<0.05). No significant difference was observed for the Otx1 protein level between the SCA3/MJD and WT group at both 12 weeks and 19 months of age (*p*>0.05).

To detect dynamic alterations in the DNA methylation status, transcription and translation of *En1*, *Otx1* and *Nkx2-1*, we performed BSP, qRT-PCR and western blot (Figure 5, Table 3 and Figure 6). For 3-month-old mice, the average DNA methylation levels of the sequences En1_2 and Otx1 were significantly higher in the SCA3/MJD group than in WT group (Figure 5A; *p*<0.05). In contrast, the average DNA methylation levels of the Nkx2-1_2 sequence were significantly lower in the SCA3/MJD group than in WT group (Figure 5A; *p*<0.05). *Otx-1* and *Nkx2-1* expression were significantly higher, and *En-1* expression was significantly lower in the SCA3/MJD group than in WT group (Figure 5B; *p*<0.05). For 19-month-old mice, the DNA methylation levels of sequences in *En1*, *Otx1* and *Nkx2-1* showed no significant difference between the two groups (Figure 5C; *p*>0.05). However, as shown in Figure 5D, *Otx1* and *Nkx2-1* expression were significantly higher in theSCA3/MJD group than in WT group. Compared to the WT group, the protein level of En1 showed a significant downregulation in SCA3/MJD group at both 12 weeks and 19 months (Figure 6; *p*<0.05). And at 19 months of age, the protein level of En1 was higher than that at 12 weeks in both WT and SCA3/MJD mice cerebellum (Figure 6; *p*<0.05). For Nkx2-1, the protein levels were significantly upregulated in the SCA3/MJD group than in WT group (Figure 6; *p*<0.05). Within the WT or SCA3/MJD group, the protein level of Nkx2-1 showed no significant difference at different time points. No significant difference was observed for the Otx1 protein level between the SCA3/MJD and WT group at both 12 weeks and 19 months of age (*p*>0.05).

**Table 3:** DNA methylation rate of sequences in *En1*, *Otx1*, and *Nkx2-1* in 12 weeks and 19 months mice between SCA3/MJD transgenic and WT groups.

**Gene**	**Sequence**	**Time**	**Group**	**Levels of DNA methylation**	***p* value**
*En1*	S1 (En1_2)	3 month	SCA3/MJD	0.222 ± 0.082	0.033*
			control	0.067 ± 0.017	
		19 month	SCA3/MJD	0.172 ± 0.009	0.286
			control	0.128 ± 0.054	
	S2 (En1_3)	3 month	SCA3/MJD	0.504 ± 0.160	0.784
			control	0.459 ± 0.210	
		19 month	SCA3/MJD	0.364 ± 0.081	0.608
			control	0.307 ± 0.192	
*Otx1*	S3 (Otx1)	3 month	SCA3/MJD	0.324 ± 0.043	0.005*
			control	0.185 ± 0.009	
		19 month	SCA3/MJD	0.164 ± 0.145	0.474
			control	0.248 ± 0.116	
*Nkx2-1*	S4 (Nkx2-1_2)	3 month	SCA3/MJD	0.063 ± 0.023	0.009*
			control	0.163 ± 0.029	
		19 month	SCA3/MJD	0.167 ± 0.093	0.239
			control	0.270 ± 0.09	
	S5 (Nkx2-1_3)	3 month	SCA3/MJD	0.656 ± 0.116	0.192
			control	0.527 ± 0.015	
		19 month	SCA3/MJD	0.622 ± 0.223	0.745

## DISCUSSION

In the present study, we investigated the DNA methylation landscape of the cerebellum of SCA3/MJD transgenic mice by RRBS analysis. To our knowledge, this represents the most systematic analysis of DNA methylation in this disease using cerebellum samples from SCA3/MJD and control mice. Although the global average methylation pattern was similar between SCA3/MJD and WT mice cerebellum at the single-CpG level, 135 DMRs were identified using this technique between the two groups, including 62 hypomethylated and 73 hypermethylated. Functional analysis indicated that these DMRs were enriched in biological pathways related to amino acid metabolism, Hedgehog signaling, thyroid cancer, tumorigenesis and other pathways. For amino acid metabolism, upregulated tryptophan 2,3-dioxygenase could upregulate the downstream compound quinolinic acid, an agonist of NMDA-R, which can increase the influx of Ca2^+^ ions that increase Ca2^+^-dependent calpain activity in SCA3/MJD [24]. Moreover, amino acid metabolism and fatty acid metabolism are related to the principal differential metabolites in symptomatic SCA3 patients compared to preclinical SCA3 patients and healthy controls [25]. Similarly, Hedgehog signaling activity in the Bergmann glia is involved in the proliferation of cerebellar granule neuron precursors and the proper maintenance of cerebellar architecture [26]. Recently there is growing evidence that polyQ disease share some similar pathogenic mechanisms with tumor such as dysregulation of DNA repair genes. For organisms, continual exposure to exogenous and endogenous stressors would lead to DNA breaks or damages [27]. So complex DNA damage repair mechanism have been developed to maintain normal physiological function in cells. Damage or improperly repaired DNA could activate pro-apoptotic pathways, give rise to oncogenic transformation, or induce cellular senescence [28, 29]. Therefore, many kinds of tumor have been found related with abnormal DNA repair mechanism. In recent years, increasing number of evidence suggested that compromised DNA repair contributed to the pathogenesis of SCA3/MJD. Distinct DNA damage has been observed in SCA3/MJD patients [30] and transgenic mice [31]. Besides this, association analysis and GWAS studies have identified DNA repair genes as modifiers of age at onset in SCA3/MJD [32, 33]. Although neither DMRs in DNA repair genes nor biological pathways related to DNA repair were found in our study, DNA methylation and transcription levels of genes in DNA repair pathway should be assessed in future study to advance our understanding of the pathogenesis of SCA3/MJD. The above results indicate that the expanded polyQ tract in ataxin3 could change the DNA methylation status of a variety of genes involved in various biological processes and, thus, cause neuronal death in the cerebellum and spinal cord due to alterations in the expression of these genes. In addition, the observed alterations in DNA methylation were gene- and sequence-dependent, consistent with previous reports, e.g., methylation levels increased in some sequences and decreased in others, even within the same gene [34].

To exclude the possibility of false positive results in the RRBS sequencing, several genes (*En1*, *Fbxo41*, *Nkx2-1*, *Syngr1*, *Rara*, *Otx1*, *Frzb*) with significantly differential methylation status and neuron-related functions were further assessed to verify their DNA methylation status and detect their expression levels by MethylTarget, qRT-PCR and western blot, respectively, in an independent cohort of 32-week-old mice. The results of the MethylTarget sequencing were consistent with the RRBS data, indicating that RRBS is an effective and practical method for exploring genome-wide DNA methylation status in SCA3/MJD mice.

Among the identified DMRs in the above 7 genes, sequences belonging to *En1*, *Otx1* and *Nkx2-1* showed dramatic changes in DNA methylation status accompanied by alterations in transcript levels. More specifically, the DMRs of *En1* and *Nkx2-1* are located in the promoter regions and their vicinity, while the DMR of Otx1 is located in an intronic region. The transcript levels of *En1* and *Nkx2-1* were inversely correlated with the DNA methylation status, while the *Otx1* transcript level showed a positive correlation with methylation level. These results are consistent with a previous report that DNA methylation in promoter regions was more thoroughly established as an epigenetic regulator of gene expression [35, 36]. The *EN1* gene encodes the homeobox protein engrailed-1. Its expression is observed within multiple neuronal cell types, including in the cerebellum [37]. To date, hypermethylation of this gene has been observed in multiple cancer types, and the extent of the hypermethylation was correlated with the grading, location and outcome of the tumor [38, 39]. However, the function of *EN1* in the central nervous system remains unclear. In our study, sequences in the promoter of *En1* were hypermethylated in SCA3/MJD mice compared to controls. This result suggested that *En1* hypermethylation might participate in the pathogenesis of both tumor and SCA3/MJD. Moreover, previous studies have shown that mitochondrial dysfunction is related to the pathogenesis of SCA3/MJD. It has been suggested that loss of heterozygosity in *EN1* may inhibit the activity of mitochondrial complex I, leading to progressive mesencephalic dopaminergic neuron degeneration in adulthood [40]. *Nkx2-1*, a homeodomain transcription factor that was initially found to regulate the transcription of many thyroid- and lung-specific genes, has been demonstrated to participate in the process of brain development in recent years [41]. It is responsible for the interneuron specification of medial ganglionic eminence cells and the regulation of guidance of migrating interneurons [42]. Knockdown of *NKX2-1* in embryonic stem cells could cause a dislocation of medial ganglion upregulation, suggesting that this gene is crucial for the differentiation of the nervous system [43]. In our study, *Nkx2-1* was found to be hypomethylated in the cerebellum of SCA3/MJD mice compared to controls. We speculated that expanded ataxin3 could influence the expression of *Nkx2-1* by changing the DNA methylation level of the promoter of this gene, inducing interneuron dysfunction. Otx1 is a transcription factor expressed in cortical progenitor cells and emerging cortical plates [44]. Mice with targeted deletion of Otx1 exhibited fewer cortical neurons and a thinner cortex than control mice [45]. In this study, the expression of Otx1 was not altered by its level of DNA methylation. Future studies should focus on further understanding the functions of these genes in the mechanism of SCA3/MJD pathogenesis.

Furthermore, this study is the first to report dynamic changes by detecting DNA methylation status, transcript and translation levels in 3-month-old and 19-month-old mice. Many studies to date have found that methylome dynamics play an important role in neuronal function [45], and it is well accepted that DNA methylation is essential for mammalian development [46]. According to our study, alteration of the target sequences’ methylation status and the transcript levels of their respective genes began as early as 3 months prior to the occurrence of ataxia symptoms. At 3 months, before we detected ataxic behavior in mice, the DNA methylation status of *En1*, *Nkx2-1*, and *Otx1* had changed, and their expression changed correspondingly. By 19 months, although there was no significant difference in DNA methylation status, the transcriptional and translational levels of *En1* and *Nkx2-1* still differed between SCA3/MJD transgenic mice and WT mice. This result suggested that DNA methylation could lead to long-term stability of phenotypes related to ataxia by regulating the permanent silencing or activation of specific genes, supporting the idea that epigenetic alterations are mitotically heritable and persistent [47–49].

Recent studies have also explored the possible cause for the difference of DNA methylation observed in polyQ disease [50]. The altered expression of DNA methyltransferases (*DNMT*) genes and DNA (de)methylation-related genes might result in the change of DNA methylation [51]. Generally speaking, DNA methylation is mainly catalyzed by DNMTs in mammals. Some studies have found that *DNMT* genes, *Gadd45a*, *Gadd45g* and *Rnf4* which involved in DNA (de)methylation were differentially expressed in HD models [52, 53]. Dnmt1 has also been found up-regulated in a spinal and bulbar muscular atrophy (SBMA) mouse model [54]. Besides this, trinucleotide repeats might be other factors which influence the level of DNA methylation [50]. And environmental factors such as diet, deficiency in essential amino acids and folate, and exposure to nicotine and other toxins have been linked to changes in global methylation changes [55–57]. All the factors mentioned above provide novel epigenetic therapeutic targets for SCA3/MJD.

In conclusion, the present study clarified the DNA methylation landscape in association with gene expression in the cerebellum of SCA3/MJD transgenic mice. We also detected dynamic alterations of DNA methylation in *En1*, *Otx1* and *Nkx2-1*. The present study identifies these genes as novel research targets regulated by DNA methylation, providing useful information for further studies of the pathogenesis of SCA3/MJD.

## MATERIALS AND METHODS

The entire experimental procedure was approved and supervised by the ethics committee of Xiangya Hospital, Central South University. The mice (B6; CBA-Tg (ATXN3*) 84.2Cce/IbezJ; ID: 012075) were purchased from Jackson Laboratory, and their offspring were used in this study. They were maintained under standard conditions (12:12 hours light-dark cycle with lights on between 06:00 and 18:00; temperature 22 ± 1° C; mouse chow and water provided ad libitum). Cerebellum samples were collected from SCA3/MJD transgenic vs. WT offspring for subsequent analysis.

PCR, agarose gel electrophoresis and capillary electrophoresis sequencing were used for the genotypic detection of the mice. The expanded CAG repeats were amplified by PCR using a pair of primers: 5’-CCAGTGACTACTTTGATTCG-3’ (forward) 5’-TGGCCTTTCACATGGATGTGAA-3’ (reverse). The amplification reaction system and conditions are summarized in Supplementary Tables 1, 2. The size of the PCR products was examined with an imaging system after 15 min of ethidium bromide (EB) staining. Capillary electrophoresis sequencing was performed to test the (CAG)n repeat number using an ABI 3730XL DNA Analyzer (Applied Biosystems, Foster City, CA, USA). The footprint and rotation tests were used to validate the phenotype of SCA3/MJD mice, as in our previous reports [58].

### DNA extraction

Adult male mice (3 transgenic and 3 WT, 32 weeks old) were sacrificed using cervical dislocation, and the cerebellums were dissected directly. Samples were immediately frozen in liquid nitrogen and stored at −80° C until use. Genomic DNA was extracted using a TIANamp Genomic DNA Kit (Tiangen, Beijing, China) according to the manufacturer’s protocol. The extracted DNA quality was evaluated by a NanoDrop 8000 Analyzer (Thermo Scientifi, USAc).

### RRBS sequencing and data analysis

RRBS sequencing was performed at BGI-Shenzhen (Shenzhen, China). First, 3 μg of total genomic DNA from each sample was digested with MspI (NEB, R0106T), and then, DNA end repair, single A nucleotide addition, and multiplexed adapter ligation were performed. The genomic DNA was purified by agarose gel electrophoresis to isolate 40–220 bp fragments. Then, a ZYMO EZ DNA Methylation-Gold™ kit (Zymo Research, D5006) was used to perform bisulfite conversion, and PCR was performed. An Agilent 2100 Bioanalyzer (Agilent Technologies, Santa Clara, CA, USA) was used to analyze the quality of the libraries, and DNA fragments with sizes ranging from 25 bp to 12,000 bp were quantified with the ABI StepOnePlus Real-Time PCR System (Thermo Fisher, USA). The resulting fragments were sequenced on an Illumina HiSeq 2000 analyzer (Illumina). After sequencing, methylation analysis of promoter and CpG island regions was performed by examining the distribution ratio of CG, CHG and CHH among the methylated C bases, the average methylation level of C bases and the distribution of different types of methylation. The methylation level and CpG density were used to calculate the regional methylation profile in a specific area, and the CpG density was defined for each CpG site within a window of 200 bp. Differentially methylated region (DMR) analysis was performed using the sliding window method to calculate the methylation differences between the SCA3/MJD and WT mouse cerebellum samples. Then, the DMRs were subjected to functional annotation, including GO and KEGG pathway analysis. For each DMR, the statistical significance of the differential methylation was calculated using Fisher’s exact test on a 2×2 contingency table of methylated and nonmethylated counts in the two groups. Multiple-hypothesis correction was applied using the Benjamini–Hochberg procedure. DMRs were identified using a final cutoff of P<1e-5.

### Validation of DNA methylation status and detection of the expression levels of the identified markers

Seven target genes with the greatest differences in DNA methylation were subjected to further analysis (Supplementary Tables 3, 4). An independent group of adult male mice (6 transgenic vs 6 WT, 32 weeks old) was used to validate the results of RRBS and to detect the expression of these differentially methylated genes. Sample acquisition, DNA extraction and preservation were performed as mentioned above.

### MethylTarget sequencing and data analysis

The genomic regions of interest were transformed to bisulfite-converted sequences using geneCpG software. Thirteen primers for the 7 target genes were designed and synthesized based on the bisulfite-converted DNA (Supplementary Table 3). Genomic DNA was subjected to sodium bisulfite treatment using the EZ DNA Methylation™-GOLD Kit (Zymo Research, Irvine, CA, USA). Multiplex PCR and index PCR were performed as described in detail elsewhere [59]. The reactions were cleaned up using the DNA Clean and Concentrator™-5, and the products were normalized by concentration and pooled. Then, the libraries were denatured, diluted, and sequenced on the Illumina MiSeq according to the manufacturer’s protocols. The sequencing run was a 150-base paired-end run. Quality control of the sequencing reads was performed by FastQC. Filtered reads were mapped to the genome by Blast after read recalibration with USEARCH. All data are presented as the mean ± SD, and t-tests or U tests were used to assess the differences between groups. Methylation and haplotypes were analyzed using Perl script. Statistical analysis was performed with t-test and ANOVA. p< 0.05 was considered statistically significant. Graphs were drawn with GraphPad Prism 5.0.

### Quantitative real-time PCR

Total RNA from mouse cerebellum samples was purified using TRIzol (Invitrogen, Carlsbad, CA, USA) and treated with DNase (from the Turbo DNA-Free kit). The RNA was reverse-transcribed for qRT-PCR assays (Maxima SYBR Green qPCR Master Mix, CFX96, Bio-Rad, Hercules, CA, USA), and each PCR was carried out in triplicate using SYBR Green PCR Master Mix (Applied Biosystems). Primers (Supplementary Table 4) were designed using Primer3 (http://bioinfo.ut.ee/primer3-0.4.0/). The qRT-PCR mixture (20 μL) consisted of a cDNA aliquot, 400 nM forward or reverse primer and 1× SYBR Green PCR Master Mix containing AmpliTaq Gold DNA polymerase and SYBR Green 1 dye. The relative expression level of the mRNA was calculated using the 2^-ΔΔCt^ method, and results with Ct >35 were excluded. PCR amplification of GAPDH mRNA was used as a control for normalization. A Wilcoxon rank sum test was used for the statistical analyses, and p< 0.05 was considered statistically significant.

### Western blot

Cerebellum tissues were lysed in a pre-cooled RIPA buffer, homogenized with ultrasound homogenizer, incubated on ice for 20min and centrifuged at 12000g for 10 mins at 4° C. The protein content was determined by the bicinchoninic acid (BCA) method (PPLYGEN, Beijing, China). Then the protein samples were subjected to various concentrations of SDS-PAGE and transferred to the PVDF membranes. The membranes were blocked with 5% non-fat dried milk in tris-buffered saline (TBS) containing 0.05% Tween-20 for 2 hour and incubate overnight with En1, Nkx2-1, or Otx1 antibody at 4° C, respectively. Then the membranes were washed 3 times with 0.1% Tween-20 TBS and incubate with anti-mouse secondary antibody for 2 h. β-actin antibody (1:2,000; Sigma-Aldrich) was used to normalize the expression data. The immunoreactive bands were visualized by enhanced chemiluminescence using Image Lab™ software with the gel imaging analysis system (Bio-Rad).

### Detection of dynamic changes in DNA methylation and gene expression in presymptomatic and aging SCA3/MJD and WT mice

*En1*, *Nkx2-1* and *Otx1*, which exhibited significant differences in DNA methylation between the two groups by both RRBS and MethylTarget sequencing, were subjected to this analysis. To observe the dynamic changes in DNA methylation and gene expression, presymptomatic mice (3 transgenic vs 3 WT at 12 weeks of age) and aging mice (3 transgenic vs 3 WT at 19 months of age) were used for bisulfite sequencing PCR (BSP), qRT-PCR and western blot analysis. Sample acquisition, DNA extraction, preservation and bisulfite modification were performed as mentioned above. The primers (Supplementary Table 5) used to amplify the DMRs were designed online with MethPrimer software (http://www.urogene.org/methprimer/). The DMRs in these genes were PCR amplified using EpiTaq™ HS reagents (Takara Bio Inc., Dalian, China). The PCRs were carried out in 50 μl reaction mixtures containing 5 μl of bisulfite-converted genomic DNA, 0.5 μl of the forward primer, 0.5 μl of the reverse primer, 6 μl of dNTPs (2.5 mM each), 5 μl of 25 mM MgCl2, 5 μl of 10 × EpiTaq PCR buffer, 0.25 μl EpiTaq HS (5 U/μl) and 27.75 μl of H2O. The PCR conditions were as follows: 98° C for 4 min; 38 cycles of 98° C for 15 s, 55° C for 30 s and 72° C for 45 s; and a final elongation at 72° C for 7 min. Then, the PCR products were separated on 2% agarose gels and purified with a gel purification kit (Sangon Biotech, Co., Ltd., Shanghai, China). The amplicons were then subcloned into the pEASY-T1 vector (Transgene Biotech, Beijing, China) according to the manufacturer’s directions for sequencing. At least 5 clones were randomly chosen for sequencing analysis using M13 forward or reverse primers (Genewiz Biotech, Beijing, China). QUMA software (http://quma.cdb.riken.jp/) was used online to analyze the sequencing results. The detection of the expression of selected genes (primers for En1, Nkx2-1 and Otx1 are displayed in Supplementary Table 4) and data analysis were performed as mentioned above.

## Supplementary Material

Supplementary Tables 1, 2, 3, 4 and 5

Supplementary Table 6

Supplementary Table 7

Supplementary Table 8

Supplementary Table 9

## References

[1] McLoughlin HS, Moore LR, Paulson HL. Pathogenesis of SCA3 and implications for other polyglutamine diseases. Pathogenesis of SCA3 and implications for other polyglutamine diseases. Neurobiol Dis. 2020; 134:104635. 10.1016/j.nbd.2019.104635 31669734 PMC6980715

[2] Seidel K, Siswanto S, Brunt ER, den Dunnen W, Korf HW, Rüb U. Brain pathology of spinocerebellar ataxias. Acta Neuropathol. 2012; 124:1–21. 10.1007/s00401-012-1000-x 22684686

[3] Dürr A, Stevanin G, Cancel G, Duyckaerts C, Abbas N, Didierjean O, Chneiweiss H, Benomar A, Lyon-Caen O, Julien J, Serdaru M, Penet C, Agid Y, Brice A. Spinocerebellar ataxia 3 and Machado-Joseph disease: clinical, molecular, and neuropathological features. Ann Neurol. 1996; 39:490–99. 10.1002/ana.410390411 8619527

[4] Kawaguchi Y, Okamoto T, Taniwaki M, Aizawa M, Inoue M, Katayama S, Kawakami H, Nakamura S, Nishimura M, Akiguchi I. CAG expansions in a novel gene for Machado-Joseph disease at chromosome 14q32.1. Nat Genet. 1994; 8:221–28. 10.1038/ng1194-221 7874163

[5] Costa Mdo C, Paulson HL. Toward understanding Machado-Joseph disease. Prog Neurobiol. 2012; 97:239–57. 10.1016/j.pneurobio.2011.11.006 22133674 PMC3306771

[6] Matos CA, de Macedo-Ribeiro S, Carvalho AL. Polyglutamine diseases: the special case of ataxin-3 and Machado-Joseph disease. Prog Neurobiol. 2011; 95:26–48. 10.1016/j.pneurobio.2011.06.007 21740957

[7] Kobayashi T, Kakizuka A. Molecular analyses of Machado-Joseph disease. Cytogenet Genome Res. 2003; 100:261–75. 10.1159/000072862 14526188

[8] Long Z, Chen Z, Wang C, Huang F, Peng H, Hou X, Ding D, Ye W, Wang J, Pan Q, Li J, Xia K, Tang B, et al. Two novel SNPs in ATXN3 3’ UTR may decrease age at onset of SCA3/MJD in Chinese patients. PLoS One. 2015; 10:e0117488. 10.1371/journal.pone.0117488 25689313 PMC4331546

[9] Peng H, Wang C, Chen Z, Sun Z, Jiao B, Li K, Huang F, Hou X, Wang J, Shen L, Xia K, Tang B, Jiang H. APOE ε2 allele may decrease the age at onset in patients with spinocerebellar ataxia type 3 or Machado-Joseph disease from the Chinese Han population. Neurobiol Aging. 2014; 35:2179.e15–18. 10.1016/j.neurobiolaging.2014.03.020 24746364

[10] Zeng L, Zhang D, McLoughlin HS, Zalon AJ, Aravind L, Paulson HL. Loss of the spinocerebellar ataxia type 3 disease protein ATXN3 alters transcription of multiple signal transduction pathways. PLoS One. 2018; 13:e0204438. 10.1371/journal.pone.0204438 30231063 PMC6145529

[11] Riley BE, Orr HT. Polyglutamine neurodegenerative diseases and regulation of transcription: assembling the puzzle. Genes Dev. 2006; 20:2183–92. 10.1101/gad.1436506 16912271

[12] Chou AH, Yeh TH, Kuo YL, Kao YC, Jou MJ, Hsu CY, Tsai SR, Kakizuka A, Wang HL. Polyglutamine-expanded ataxin-3 activates mitochondrial apoptotic pathway by upregulating Bax and downregulating Bcl-xL. Neurobiol Dis. 2006; 21:333–45. 10.1016/j.nbd.2005.07.011 16112867

[13] Chou AH, Yeh TH, Ouyang P, Chen YL, Chen SY, Wang HL. Polyglutamine-expanded ataxin-3 causes cerebellar dysfunction of SCA3 transgenic mice by inducing transcriptional dysregulation. Neurobiol Dis. 2008; 31:89–101. 10.1016/j.nbd.2008.03.011 18502140

[14] Raposo M, Bettencourt C, Maciel P, Gao F, Ramos A, Kazachkova N, Vasconcelos J, Kay T, Rodrigues AJ, Bettencourt B, Bruges-Armas J, Geschwind D, Coppola G, Lima M. Novel candidate blood-based transcriptional biomarkers of Machado-Joseph disease. Mov Disord. 2015; 30:968–75. 10.1002/mds.26238 25914309

[15] Portela A, Esteller M. Epigenetic modifications and human disease. Nat Biotechnol. 2010; 28:1057–68. 10.1038/nbt.1685 20944598

[16] Zhai D, Li S, Dong G, Zhou D, Yang Y, Wang X, Zhao Y, Yang Y, Lin Z. The correlation between DNA methylation and transcriptional expression of human dopamine transporter in cell lines. Neurosci Lett. 2018; 662:91–97. 10.1016/j.neulet.2017.10.013 29030220

[17] Chuang YH, Paul KC, Bronstein JM, Bordelon Y, Horvath S, Ritz B. Parkinson’s disease is associated with DNA methylation levels in human blood and saliva. Genome Med. 2017; 9:76. 10.1186/s13073-017-0466-5 28851441 PMC5576382

[18] Imm J, Kerrigan TL, Jeffries A, Lunnon K. Using induced pluripotent stem cells to explore genetic and epigenetic variation associated with Alzheimer’s disease. Epigenomics. 2017; 9:1455–68. 10.2217/epi-2017-0076 28969469

[19] Laffita-Mesa JM, Bauer PO, Kourí V, Peña Serrano L, Roskams J, Almaguer Gotay D, Montes Brown JC, Martínez Rodríguez PA, González-Zaldívar Y, Almaguer Mederos L, Cuello-Almarales D, Aguiar Santiago J. Epigenetics DNA methylation in the core ataxin-2 gene promoter: novel physiological and pathological implications. Hum Genet. 2012; 131:625–38. 10.1007/s00439-011-1101-y 22037902

[20] Libby RT, Hagerman KA, Pineda VV, Lau R, Cho DH, Baccam SL, Axford MM, Cleary JD, Moore JM, Sopher BL, Tapscott SJ, Filippova GN, Pearson CE, La Spada AR. CTCF cis-regulates trinucleotide repeat instability in an epigenetic manner: a novel basis for mutational hot spot determination. PLoS Genet. 2008; 4:e1000257. 10.1371/journal.pgen.1000257 19008940 PMC2573955

[21] Ding D, Wang C, Chen Z, Peng H, Li K, Zhou X, Peng Y, Wang P, Hou X, Li T, Qiu R, Xia K, Sequeiros J, et al. Polymorphisms in DNA methylation-related genes are linked to the phenotype of Machado-Joseph disease. Neurobiol Aging. 2019; 75:225.e1–8. 10.1016/j.neurobiolaging.2018.11.002 30554804

[22] Wang C, Peng H, Li J, Ding D, Chen Z, Long Z, Peng Y, Zhou X, Ye W, Li K, Xu Q, Ai S, Song C, et al. Alteration of methylation status in the ATXN3 gene promoter region is linked to the SCA3/MJD. Neurobiol Aging. 2017; 53:192.e5–10. 10.1016/j.neurobiolaging.2016.12.014 28094059

[23] Cemal CK, Carroll CJ, Lawrence L, Lowrie MB, Ruddle P, Al-Mahdawi S, King RH, Pook MA, Huxley C, Chamberlain S. YAC transgenic mice carrying pathological alleles of the MJD1 locus exhibit a mild and slowly progressive cerebellar deficit. Hum Mol Genet. 2002; 11:1075–94. 10.1093/hmg/11.9.1075 11978767

[24] Rajamani K, Liu JW, Wu CH, Chiang IT, You DH, Lin SY, Hsieh DK, Lin SZ, Harn HJ, Chiou TW. N-butylidenephthalide exhibits protection against neurotoxicity through regulation of tryptophan 2, 3 dioxygenase in spinocerebellar ataxia type 3. Neuropharmacology. 2017; 117:434–46. 10.1016/j.neuropharm.2017.02.014 28223212

[25] Yang ZH, Shi CH, Zhou LN, Li YS, Yang J, Liu YT, Mao CY, Luo HY, Xu GW, Xu YM. Metabolic profiling reveals biochemical pathways and potential biomarkers of spinocerebellar ataxia 3. Front Mol Neurosci. 2019; 12:159. 10.3389/fnmol.2019.00159 31316347 PMC6611058

[26] Cheng FY, Fleming JT, Chiang C. Bergmann glial sonic hedgehog signaling activity is required for proper cerebellar cortical expansion and architecture. Dev Biol. 2018; 440:152–66. 10.1016/j.ydbio.2018.05.015 29792854 PMC6014626

[27] Madabhushi R, Pan L, Tsai LH. DNA damage and its links to neurodegeneration. Neuron. 2014; 83:266–82. 10.1016/j.neuron.2014.06.034 25033177 PMC5564444

[28] Iyama T, Wilson DM 3rd. DNA repair mechanisms in dividing and non-dividing cells. DNA Repair (Amst). 2013; 12:620–36. 10.1016/j.dnarep.2013.04.015 23684800 PMC3720834

[29] Jackson SP, Bartek J. The DNA-damage response in human biology and disease. Nature. 2009; 461:1071–78. 10.1038/nature08467 19847258 PMC2906700

[30] Chatterjee A, Saha S, Chakraborty A, Silva-Fernandes A, Mandal SM, Neves-Carvalho A, Liu Y, Pandita RK, Hegde ML, Hegde PM, Boldogh I, Ashizawa T, Koeppen AH, et al. The role of the mammalian DNA end-processing enzyme polynucleotide kinase 3'-phosphatase in spinocerebellar ataxia type 3 pathogenesis. PLoS Genet. 2015; 11:e1004749. 10.1371/journal.pgen.1004749 25633985 PMC4310589

[31] Kazachkova N, Raposo M, Montiel R, Cymbron T, Bettencourt C, Silva-Fernandes A, Silva S, Maciel P, Lima M. Patterns of mitochondrial DNA damage in blood and brain tissues of a transgenic mouse model of Machado-Joseph disease. Neurodegener Dis. 2013; 11:206–14. 10.1159/000339207 22832131

[32] Bettencourt C, Hensman-Moss D, Flower M, Wiethoff S, Brice A, Goizet C, Stevanin G, Koutsis G, Karadima G, Panas M, Yescas-Gómez P, García-Velázquez LE, Alonso-Vilatela ME, et al, and SPATAX Network. DNA repair pathways underlie a common genetic mechanism modulating onset in polyglutamine diseases. Ann Neurol. 2016; 79:983–90. 10.1002/ana.24656 27044000 PMC4914895

[33] Wang C, Chen Z, Peng H, Peng Y, Zhou X, Yang H, Wang P, Li T, Hou X, Qiu R, Xia K, Sequeiros J, Tang B, Jiang H. Investigation on modulation of DNA repair pathways in Chinese MJD patients. Neurobiol Aging. 2018; 71:267.e5–6. 10.1016/j.neurobiolaging.2018.06.024 30033072

[34] Luo L, Yao Z, Ye J, Tian Y, Yang C, Gao X, Song M, Liu Y, Zhang Y, Li Y, Zhang X, Fang F. Identification of differential genomic DNA methylation in the hypothalamus of pubertal rat using reduced representation bisulfite sequencing. Reprod Biol Endocrinol. 2017; 15:81. 10.1186/s12958-017-0301-2 28985764 PMC5639587

[35] Seelan RS, Mukhopadhyay P, Pisano MM, Greene RM. Effects of 5-aza-2'-deoxycytidine (decitabine) on gene expression. Drug Metab Rev. 2018; 50:193–207. 10.1080/03602532.2018.1437446 29455551

[36] Qureshi IA, Mehler MF. Epigenetic mechanisms underlying nervous system diseases. Handb Clin Neurol. 2018; 147:43–58. 10.1016/B978-0-444-63233-3.00005-1 29325627 PMC6822391

[37] Wilson SL, Kalinovsky A, Orvis GD, Joyner AL. Spatially restricted and developmentally dynamic expression of engrailed genes in multiple cerebellar cell types. Cerebellum. 2011; 10:356–72. 10.1007/s12311-011-0254-5 21431469 PMC3170510

[38] Carrascosa LG, Sina AA, Palanisamy R, Sepulveda B, Otte MA, Rauf S, Shiddiky MJ, Trau M. Molecular inversion probe-based SPR biosensing for specific, label-free and real-time detection of regional DNA methylation. Chem Commun (Camb). 2014; 50:3585–88. 10.1039/c3cc49607d 24567954

[39] Devaney J, Stirzaker C, Qu W, Song JZ, Statham AL, Patterson KI, Horvath LG, Tabor B, Coolen MW, Hulf T, Kench JG, Henshall SM, Pe Benito R, et al. Epigenetic deregulation across chromosome 2q14.2 differentiates normal from prostate cancer and provides a regional panel of novel DNA methylation cancer biomarkers. Cancer Epidemiol Biomarkers Prev. 2011; 20:148–59. 10.1158/1055-9965.EPI-10-0719 21098650

[40] Yoon BC, Jung H, Dwivedy A, O’Hare CM, Zivraj KH, Holt CE. Local translation of extranuclear lamin B promotes axon maintenance. Cell. 2012; 148:752–64. 10.1016/j.cell.2011.11.064 22341447 PMC3314965

[41] Alvarez-Fischer D, Fuchs J, Castagner F, Stettler O, Massiani-Beaudoin O, Moya KL, Bouillot C, Oertel WH, Lombès A, Faigle W, Joshi RL, Hartmann A, Prochiantz A. Engrailed protects mouse midbrain dopaminergic neurons against mitochondrial complex I insults. Nat Neurosci. 2011; 14:1260–66. 10.1038/nn.2916 21892157

[42] Butt SJ, Sousa VH, Fuccillo MV, Hjerling-Leffler J, Miyoshi G, Kimura S, Fishell G. The requirement of Nkx2-1 in the temporal specification of cortical interneuron subtypes. Neuron. 2008; 59:722–32. 10.1016/j.neuron.2008.07.031 18786356 PMC2562525

[43] Minocha S, Valloton D, Arsenijevic Y, Cardinaux JR, Guidi R, Hornung JP, Lebrand C. Nkx2.1 regulates the generation of telencephalic astrocytes during embryonic development. Sci Rep. 2017; 7:43093. 10.1038/srep43093 28266561 PMC5339799

[44] Figueira Muoio VM, Uno M, Oba-Shinjo S, da Silva R, Araújo Pereira BJ, Clara C, Matushita H, Marie SNK. OTX1 and OTX2 Genes in Medulloblastoma. World Neurosurg. 2019; 127:e58–e64. 10.1016/j.wneu.2019.02.013 30797919

[45] Huang B, Li X, Tu X, Zhao W, Zhu D, Feng Y, Si X, Chen JG. OTX1 regulates cell cycle progression of neural progenitors in the developing cerebral cortex. J Biol Chem. 2018; 293:2137–48. 10.1074/jbc.RA117.001249 29273633 PMC5808773

[46] Smith ZD, Meissner A. DNA methylation: roles in mammalian development. Nat Rev Genet. 2013; 14:204–20. 10.1038/nrg3354 23400093

[47] Kaushal A, Zhang H, Karmaus WJ, Everson TM, Marsit CJ, Karagas MR, Tsai SF, Wen HJ, Wang SL. Genome-wide DNA methylation at birth in relation to in utero arsenic exposure and the associated health in later life. Environ Health. 2017; 16:50. 10.1186/s12940-017-0262-0 28558807 PMC5450181

[48] Cardenas A, Koestler DC, Houseman EA, Jackson BP, Kile ML, Karagas MR, Marsit CJ. Differential DNA methylation in umbilical cord blood of infants exposed to mercury and arsenic in utero. Epigenetics. 2015; 10:508–15. 10.1080/15592294.2015.1046026 25923418 PMC4622995

[49] Alvarado S, Rajakumar R, Abouheif E, Szyf M. Epigenetic variation in the egfr gene generates quantitative variation in a complex trait in ants. Nat Commun. 2015; 6:6513. 10.1038/ncomms7513 25758336

[50] Liu H, Tang TS, Guo C. Epigenetic profiles in polyglutamine disorders. Epigenomics. 2018; 10:9–25. 10.2217/epi-2017-0089 29172694

[51] Klose RJ, Bird AP. Genomic DNA methylation: the mark and its mediators. Trends Biochem Sci. 2006; 31:89–97. 10.1016/j.tibs.2005.12.008 16403636

[52] Jia H, Morris CD, Williams RM, Loring JF, Thomas EA. HDAC inhibition imparts beneficial transgenerational effects in Huntington’s disease mice via altered DNA and histone methylation. Proc Natl Acad Sci USA. 2015; 112:E56–64. 10.1073/pnas.1415195112 25535382 PMC4291662

[53] Narayanan M, Huynh JL, Wang K, Yang X, Yoo S, McElwee J, Zhang B, Zhang C, Lamb JR, Xie T, Suver C, Molony C, Melquist S, et al. Common dysregulation network in the human prefrontal cortex underlies two neurodegenerative diseases. Mol Syst Biol. 2014; 10:743. 10.15252/msb.20145304 25080494 PMC4299500

[54] Kondo N, Tohnai G, Sahashi K, Iida M, Kataoka M, Nakatsuji H, Tsutsumi Y, Hashizume A, Adachi H, Koike H, Shinjo K, Kondo Y, Sobue G, Katsuno M. DNA methylation inhibitor attenuates polyglutamine-induced neurodegeneration by regulating Hes5. EMBO Mol Med. 2019; 11:e8547. 10.15252/emmm.201708547 30940675 PMC6505579

[55] Richmond RC, Simpkin AJ, Woodward G, Gaunt TR, Lyttleton O, McArdle WL, Ring SM, Smith AD, Timpson NJ, Tilling K, Davey Smith G, Relton CL. Prenatal exposure to maternal smoking and offspring DNA methylation across the lifecourse: findings from the avon longitudinal study of parents and children (ALSPAC). Hum Mol Genet. 2015; 24:2201–17. 10.1093/hmg/ddu739 25552657 PMC4380069

[56] Bellavia A, Urch B, Speck M, Brook RD, Scott JA, Albetti B, Behbod B, North M, Valeri L, Bertazzi PA, Silverman F, Gold D, Baccarelli AA. DNA hypomethylation, ambient particulate matter, and increased blood pressure: findings from controlled human exposure experiments. J Am Heart Assoc. 2013; 2:e000212. 10.1161/JAHA.113.000212 23782920 PMC3698788

[57] Poirier LA. The effects of diet, genetics and chemicals on toxicity and aberrant DNA methylation: an introduction. J Nutr. 2002; 132:2336S–9S. 10.1093/jn/132.8.2336S 12163688

[58] Long Z, Li T, Chen Z, Peng Y, Wang C, Hou X, Yuan H, Wang P, Xie Y, He L, Zhou X, Peng H, Qiu R, et al. Cerebellar lncRNA expression profile analysis of SCA3/MJD mice. Int J Genomics. 2018; 2018:5383517. 10.1155/2018/5383517 30046585 PMC6036799

[59] Wang X, Tang D, Shen P, Xu H, Qiu H, Wu T, Gao X. Analysis of DNA methylation in chondrocytes in rats with knee osteoarthritis. BMC Musculoskelet Disord. 2017; 18:377. 10.1186/s12891-017-1739-2 28859619 PMC5579940

