# Supplementary material for: Polyglutamine-expanded ataxin3 alter specific gene expressions through changing DNA methylation status in SCA3/MJD
Source: Aging (Albany NY). 2020 Dec 19;13(3):3680–98. doi: 10.18632/aging.202331 (PMC7906150; doi:10.18632/aging.202331)
Supplement: Supplementary Tables 1, 2, 3, 4 and 5 [file aging-13-202331-s001.pdf]

SUPPLEMENTARY TABLES

Supplementary Table 1. The amplification reaction system of genotyping of mice.

Reagents	Volume
r Taq DNA polymerase	0.2 µl
10×PCR buffer	1 µl
DNA	1 µl
dNTP	0.2 µl
Primer-Forward (100 ng/ul)	0.2 µl
Primer-Reverse (100 ng/ul)	0.2 µl
ddH_2O	7.2 µl

Supplementary Table 2. The amplification reaction condition of genotyping of mice.

Cycle	Temperature	Time
1	94° C	5 min
	94° C	30 s
38	59° C	30 s
	72° C	15 s
1	72° C	10 min
1	4° C	Forever

Supplementary Table 3. Primers in methyltarget sequencing for validation of DNA methylation status.

Primers	Sequence
En1_1_F	GATAGGTTTAGTTAGTGATTTGGTTTTG
En1_1_R	ACCTTTCTAACTAAAAYCTTCCTCTACATTC
En1_2_F	GGAGAGGTTTTTGTTATTAAGTTTTAGTTTAG
En1_2_R	CAACCAAACCCACCAATCTAAC
En1_3_F	TATTYGGGAAAAGAGGAAATGT
En1_3 _R	AAAACCTCTATATTAAATTTCCCTACAAA
Fbxo41_F	TTTAGTYGTTTTAGTTTTYGGTTTATTTTTAG
Fbxo41_R	AAAAACTAACTAAACCDAAACTCRTACC
Frzb_1_F	GTTATTGTTTTTAGGAGGTTGTTATTTTTG
Frzb_1_R	CACACCTTTCCCCACTCC
Frzb_2_F	TTATTAAGTTTTGTAAGTTTGTGTGTGAG
Frzb_2_R	ACAAACTAACCRATAAAAACAAATAACTAAC
Nkx2-1_1_F	AGTAAGTGTATTTTTGTATGGTTGAGTTT
Nkx2-1_1_R	CCCAACACCCCAACTCAA
Nkx2-1_2_F	GGTAATTTGGGTAATATGAGYGAGTTGT
Nkx2-1_2_R	CAATACCCTCACCCCACTCC
Nkx2-1_3_F	TTGGTGGTTTGGGTGTGTT
Nkx2-1_3_R	CAACAACAACAAACTCAACAACAA
Rara_1_F	TGGGAAGGTGGTATTATTTTGG
Rara_1_R	CACACTTCTAATTCTCCACCTAAAA
Rara_2_F	AATTGTTAYGAGTGAAGGTTAAGAGG
Rara_2_R	ACAAAACAAATCTATTCCTAAAACCATC
Syngr1_F	GGTGGTTTTGGAGTAGGTTTTTTT
Syngr1_R	AACCCAAAAACAACCCTCTAAAC
Otx1_F	TTCGYGTTATTTTGTAGGTTTTTAGTTTT
Otx1_R	AACCRAAAATAATAACAACCTAATCCT

Supplementary Table 4. Primers in qRT-PCR for detection of gene transcription.

Gene	Primers	Product size
GAPDH	F: 5' GGTTGTCTCCTGCGACTTCA 3'<br>R: 5' TGGTCCAGGGTTTCTTACTCC 3'	183bp
Otx1	F: 5' ACCTTCCTTCTCCGAAATCTG 3'<br>R: 5' GGACTGCCTTACATCTCAACAA 3'	149bp
En1	F: 5' AAGAACGAGAAGGAAGACAAGC 3'<br>R: 5' ATATAGCGGTTTGCCTGGAA 3'	92 bp
Nkx2-1	F: 5' CGAGATATTCGCCTCCTCCC 3'<br>R: 5' GATTCGGCGTCGGCTGG 3'	248bp
Frzb	F: 5' ATGATGTGACCGCCGTTGT 3'<br>R: 5' CGTTCCTCGTCTTCATAGCC 3'	160bp
Syngr1	F: 5' CAGTGTCAAGGACCGCAAGA 3'<br>R: 5' GGAAGCAGAAACCCACGAAC 3'	89 bp
Fbxo41	F: 5' TCTTCTGTATCTTCACCTACCTGG 3'<br>R: 5' TGGAACAGACTCGGGCATT 3'	135bp
Rara	F: 5' CAGTGCCATCTGCCTCATCT 3'<br>R: 5' CATCAGCATCTTGGGGAACAT 3'	151bp

Supplementary Table 5. Primers in BSP for detection of DNA methylation status in 3 months and 19 months mice.

Primers		Primers sequence	Product size
En1-2	F	AGAAGTTTTAGAAAGTTGGAGAGAT	391bp
	R	TTTCCCACCCAATATCCTATTAAA	
En1-3	F	ATATTGGGTGGGAAAGGTTTT	443bp
	R	AATATACCTCCTTACAAAACCTAAAT	
Otx1	F	GGTGGTATTTAAGATTAATTTGTTAG	671bp
	R	ATTACATTCCCAAACCCTTCTC	
Nkx2-1-1	F	GTAGGTTAAGATTTGGTTTTAGA	429bp
	R	AAAACCTAACCCTACAACCC	
Nkx2-1-2	F	TTGGTAGGTTGATTTTGAGTGT	462bp
	R	CCTATTTCTATCCACCTCTACTCTA	
